# Artifacts among Cone Beam Computed Tomography Images of Patients of Tertiary Care Centre: A Descriptive Cross-sectional Study

**DOI:** 10.31729/jnma.7949

**Published:** 2023-01-31

**Authors:** Harleen Bali, Abhinaya Luitel, Chandan Upadhyaya

**Affiliations:** 1 Department of Oral Medicine and Radiology, Kathmandu University School of Medical Sciences, Dhulikhel, Kavrepalanchowk, Nepal; 2 Department of Oral and Maxillofacial Surgery, Kathmandu University School of Medical Sciences, Dhulikhel, Kavrepalanchowk, Nepal

**Keywords:** *artefact*, *cone beam computed tomography*, *radiation*

## Abstract

**Introduction:**

Cone beam computed tomography is widely used as a mode of investigation in the field of dentistry. Although presenting a three-dimensional picture of head and neck structures it does carry drawbacks in the form of artifacts which not only degrade image quality but a repeat of the radiograph leading the patient to radiation exposure again. This study aimed to find out the prevalence of artifacts among cone beam computed tomography images of patients visiting tertiary care centre.

**Methods:**

A descriptive cross-sectional study was conducted on cone beam computed tomography images of patients in the archives of dental radiology at the Department of Oral Medicine and Radiology wherein all cone beam computed tomography radiographs of patients after taking an ethical approval from Institutional Review Committee (Approval number: 13/22) from 1 January 2019 to 19 March 2022 were included in the study. The study included 780 image of patients. Convenience sampling was used. The artifacts when present was noted and categorised as inherent artifacts, procedure-related artifacts, introduced artifacts and patient motion artifacts. Point estimate and 95% Confidence Interval were calculated.

**Results:**

Among 780 cone beam computed tomography image patients, artifacts were seen in 665 (85.25%) (82.76-87.74, 95% Confidence Interval) study images.

**Conclusions:**

The prevalence of artifacts among cone beam computed tomography images of patients is similar to the studies done in similar settings.

## INTRODUCTION

Cone beam computed tomography (CBCT) has become an integral part of the investigation of the oral maxillofacial region. With lower radiation dose compared to conventional computed tomography (CT)^1^ and 3-Dimensional information over 2-Dimensional extra-oral radiographs, their application in dentistry has increased tremendously.

However, can seriously degrade the quality of CBCT images, sometimes to the point of making them diagnostically unusable. White and Pharoah define an artifact as any distortion or error in the image that is unrelated to the subject being studied.^2^ Knowledge of these can prevent repeated exposure of the patient. To the best of our knowledge and literature search, we could not find any study on the artifact in CBCT from Nepal to date.

Hence this study aimed to find out the prevalence of among cone beam computed tomography images of patients visiting tertiary care centre.

## METHODS

A descriptive cross-sectional study was conducted on CBCT images of patients in the archives of dental radiology at the Department of Oral Medicine and Radiology, Dhulikhel hospital wherein all CBCT radiographs of patients taken from 1 January 2019 to 19 March 2022 were included in the study. Ethical clearance was obtained from the Institutional Review Committee of the hospital (Approval number: 13/22). The sample size was calculated using the following formula:


$$\begin{array}{ll} \text{n} & ={\text{Z}}^{2}\times \frac{\text{p}\times \text{q}}{{\text{e}}^{\text{2}}}\\ & =1.96^{2}\times \frac{0.50\times 0.50}{0.05^{2}}\\ & =385 \end{array}$$

Where,

n = minimum required sample size
Z = 1.96 at 95% of Confidence Interval (CI)
p = prevalence is taken as 50% for maximum sample size calculation
q = 1-p
e = margin of error, 5%

The sample size was calculated to be 385 radiographs. The inclusion criteria were CBCT images in the archives of dental radiology. Exclusion criteria are blank or no images seen for the recorded scan. Thereby 780 CBCT images were included in the study, using the convenience sampling method. The images were taken by Dentium Rainbow CBCT machine having parameters such as scan Time: 20 seconds, peak Voltage: 100 kVp, tube Current: 12 mA, Field of View: 16x18 cm^2^ and voxel size: 300 μm. Volume CT data was acquired. Multi-planar reconstruction was performed on a viewing workstation to obtain axial and coronal images. The obtained images were viewed and analyzed in Rainbow TM Image Viewer Version 1.0.0.0. The images were viewed on the same computer screen using the same image viewer, under ambient light with all curtains closed by an oral radiologist with more than three years of experience in CBCT reporting. The artifact when present was noted and categorised as Inherent, procedure-related, introduced and patient motion.^3^ Inherent are a result of the Cone beam projection geometry of CBCT. They are three types:

- Scatter-causes overall image degradation or quantum noise
- Partial volume averaging-it results in boundaries having a step appearance
- Cone-beam effect-image has greater peripheral noise and image distortion

Aliasing is a type of procedure-related artifact which presents as fine striations in the scanned images. Ring or circular streaks and double contours are the other two types of procedure-related.

Introduced which include the phenomenon of beam hardening resulting in

- Cupping-seen as a distortion of metallic structures
- Streaks and dark bands

Double contours of the image are a result of patient motion artifact.

The data were recorded in a Microsoft Excel 2014 and analyzed using IBM SPSS Statistics version 20.0. Point estimate and 95% CI were calculated.

## RESULTS

Among 780 cone beam computed tomography image patients, were seen in 665 (85.25%) (82.76-87.74, 95% Confidence Interval) study images of patients. All the radiographs having had more than one artifact present. There was a total of 665 (38.08%) inherent in the form of noise and scattering, along with 665 (38.08%) procedure-related as in the Aliasing effect. All 373 (56.09%) CBCT images of patients having metal objects in area to be scanned had introduced. Double contours due to patient motion made up 43 (2.46%). The were more pronounced in segmental images rather than in maxillofacial or craniofacial images. The frequency of occurrence of CBCT (Table 1).

**Table 1:** Classification and occurrence of in CBCT images (n= 665).

Type of artifacts	n (%)	n (%)
Inherent artifact	665 (100)	665 (100)
Step appearance	-	
Cone-beam effect	-	
**Procedure-related artifact**		
Aliasing	665 (100)	665 (100)
Ring or circular streaks	-	
**Introduced**		
Dark bands	4 (1.07)	373 (56.09)
Streaks and dark bands	40 (10.72)	
Cupping, streaks and dark bands	329 (56.09)	
**Patient motion**	43 (100)	43 (6.47)

## DISCUSSION

Two-dimensional radiographs provide limited information due to superimposition and distortions. Cone beam CT (CBCT) since its introduction in 1998 in Italy has been widely used in the diagnosis and management of oro-maxillofacial anomalies.^3^ Even then CBCT has its limitations such as artifact. Artifact are responsible for retakes of radiographs leading to unnecessary patient exposure to radiation. A minimum of 95% of images is required to be diagnostically acceptable in order to avoid retakes as recommended by the Health Protection Agency guidelines for dental CBCT scans.^4^ Keeping the Alara principle in mind which states that radiation doses to patients and personnel be as low as reasonably achievable^5^ it becomes a necessity for the operator and clinician to be aware of reasons for the production of and ways to avoid and reduce them.

In our study, the majority of images had which was similar to the study done in Turkey in 2019 wherein they studied 600 CBCT images and found only 15 images without any.^6^ Whereas a study in 2019 from India found that out of 900 CBCT images only 42 were repeated because of artifacts.^7^ In the present study, inherent in the form of noise and scatter, and aliasing which is procedure-related was noted as the most common artifact.

In general, poor calibration or difficulties in scanner detection led to in 9% of radiographs that were repeated in a study done in 2018 in Germany.^8^ In 2019 in India, studied a total of 42 images had , of these 10 (23.81%) had noise but none of the images showed aliasing or scatters.^7^

Scatter is caused when photons diffract from their path after interaction with matter. Flat panel detector (FPD) technology, used in CBCT, on one side provides exceptional spatial resolution with a comparatively low patient radiation exposure but on the other hand contrast resolution is affected adversely due to increased X-ray scatter and reduced temporal resolution. This leads to a reduction in low contrast resolution making it difficult to differentiate low-density tissues and their boundary in the resultant image. Conventional CT machines use high mA and pre and post-patient collimation which reduce the scattered radiation to a negligible amount as compared to CBCT machines where the noise is more due to the use of lower mA and soaring scattered radiation from the absence of post-patient collimation.^9^

In Turkey in 2019 studied 600 CBCT images were studied. Of these, they found 348 images (23.1% of total) to show an aliasing effect and that aliasing artifact is highly and positively correlated with acquisition time.^6^ In case few basis projections are made available for image reconstruction or rotational trajectory arcs are not completed, undersampling of the imaged object occurs, leading to misregistration, sharp edges, and noisier images which are resultant of the aliasing effect. Then again increasing the number of basis projections or a complete arc rotation can increase the exposure of the patient to radiation.^2^

In the present study, all of the images having metal objects showed beam hardening in form of streaks, bands, and cupping. A study done in Chennai India reported that beam hardening or streak constituted 7.14% of the total in their study.^7^ Metal showed a statistically significant association with the field of view (FOV) and acquisition time in an Italian study in 2015 in 500 CBCT images. They observed that in large FOVs artifact is not seen because it includes regions away from the site of metal objects i.e. jaws.^10^ In a study done in Turkey, beam hardening (dark band or streaks) was the most common artifact (585 out of 600 images), though the association between both metal and FOV or acquisition times was not statistically significant.^6^

Beam hardening artifact is a type of introduced artifact and is seen because the average energy of the beam increases as the lower energy photons are absorbed in comparison to higher energy photons in the presence of a metal object in the path of radiation. This presents two types of artifact-cupping and extinction or missing-value. Cupping artifact occurs when x-rays passing through the centre of a metal object become harder than those passing through the edges of the object due to the greater amount of material the beam has to penetrate it appears as a "cup".^11^ Extinction or missing value artifact is seen as streaks and dark bands between two metal objects where deformation of metallic structure occurs due to differential absorption.^2^

Reducing Field of View (FOV), modifying patient position or separating dental arches to avoid regions with non-removable metal objects, and advising patients to remove metal jewellery from the head and neck region before scanning are some ways to avoid beam hardening from occurring in the region of interest (ROI).^2^ A study in 2011 in Japan observed that increase in kVp resulted in a decrease in whereas an increase in tube electric current showed no effect on.^12^ Furthermore beam hardening can be reduced by using filtration, anti-scatter grids, calibration correction, and beam hardening correction software.^13^

In the present study, we found it to be 2.46% in the form of double contours. Patient motion have been reported before between 4.5% and 48.2%. CBCT images of younger and older age groups had higher chances of motion artifact as compared to the middle age group observed in many studies.^10,14-16^ A study done in Turkey concluded that motion artifact was significantly higher in images taken in standing position, however, no correlation was observed in sitting and supine positions.^16^

In 2014 Brazil compared the dental plaster model and cone-beam computed tomography image in the measurement of the dental arches and investigated whether CBCT image can compromise the reliability of such measurements. They noted that CBCT images were negatively influenced by the presence of image.^17^ Therefore, the need to reduce in order to maintain image quality is vital to diagnosis and treatment planning.

The limitation of the present study was that study did not assess if the type of metal density affected the resultant beam hardening artifact. It did not account for whether the distance of the metal object from the Region of Interest (ROI) is relevant. Also, further studies employing a larger sample size and comparing CBCT machines of different make are recommended by the authors.

## CONCLUSIONS

The prevalence of among cone beam computed tomography images of patients is similar to the studies done in similar settings. Our study reported scatter and aliasing as the most common artifact, followed by beam hardening. can be caused by several factors which ultimately degrade the quality of CBCT images to diverse degrees. Therefore a way to reduce them is not only by the operator and patient being aware but by making use of technology such as artifact-reducing software and decreasing acquisition time. The aim remains to provide patients with the best treatment opportunity.
